# A role for intracellular zinc in glioma alteration of neuronal chloride equilibrium

**DOI:** 10.1038/cddis.2014.437

**Published:** 2014-10-30

**Authors:** S Di Angelantonio, E Murana, S Cocco, F Scala, C Bertollini, M G Molinari, C Lauro, P Bregestovski, C Limatola, D Ragozzino

**Affiliations:** 1 Istituto Pasteur-Fondazione Cenci Bolognetti, Department of Physiology and Pharmacology, Sapienza University of Rome, Piazzale Aldo Piazzale Aldo Moro 5, Roma 00185, Italy; 2 Center for Life Nano Science@Sapienza, Istituto Italiano di Tecnologia, Viale Regina Elena 291, Roma 00161, Italy; 3 INSERM URM 1106, Aix-Marseille University, Brain Dynamics Institute, Marseille, France; 4 IRCCS Neuromed, Via Atinese, Pozzilli, Italy

## Abstract

Glioma patients commonly suffer from epileptic seizures. However, the mechanisms of glioma-associated epilepsy are far to be completely understood. Using glioma-neurons co-cultures, we found that tumor cells are able to deeply influence neuronal chloride homeostasis, by depolarizing the reversal potential of *γ*-aminobutyric acid (GABA)-evoked currents (E_GABA_). E_GABA_ depolarizing shift is due to zinc-dependent reduction of neuronal KCC2 activity and requires glutamate release from glioma cells. Consistently, intracellular zinc loading rapidly depolarizes E_GABA_ in mouse hippocampal neurons, through the Src/Trk pathway and this effect is promptly reverted upon zinc chelation. This study provides a possible molecular mechanism linking glioma invasion to excitation/inhibition imbalance and epileptic seizures, through the zinc–mediated disruption of neuronal chloride homeostasis.

Glioma-associated epilepsy is an established but poorly understood phenomenon. Over 80% of glioma patients suffer from seizures,^1^ often representing the first symptomatic presentation of a tumor and possibly preceding it.^2^ It has been extensively reported that glioma cells release glutamate in the extracellular space, through the glutamate-cystine transporter (system Xc), promoting proliferation and invasion and causing neuronal death.^3^ Accordingly, increased glutamate levels have been implicated in numerous seizure disorders^4^ and contribute to epileptogenesis in glioma-implanted rodents.^5, 6^ Glutamate excess may cause the alteration of neuronal chloride (Cl^−^) homeostasis and depolarize *γ*-aminobutyric acid (GABA) reversal potential (E_GABA_), as a result of Cl^−^ transporters dysfunction or disequilibrium.^7^ Indeed, a precise balance between NKCC1 and KCC2 activity is necessary for inhibitory GABAergic signaling in the adult central nervous system^8^ and its disequilibrium can cause elevation of intracellular [Cl^−^] leading to switch of GABAergic signaling from hyperpolarizing to depolarizing in epileptic tissue,^9, 10^ contributing to epileptogenesis.^11, 12, 13^

In this study, we investigated the mechanisms of glioma-induced neuronal overexcitation using co-cultures of hippocampal and glioma cells. We report that glioma cells cause the alteration of E_GABA_, through glutamate-receptor-dependent zinc (Zn^2+^) accumulation, leading to KCC2-mediated Cl^−^ transport unbalance. Our study provides the molecular mechanism of glioma-induced elevation in intracellular Cl^−^ and a complete model linking glutamate release by glioma cells to glioma-related epilepsy.

## Results

### Glioma co-culture increases neuronal [Cl^-^]_i_ by a glutamatergic mechanism

**Figure 1:**
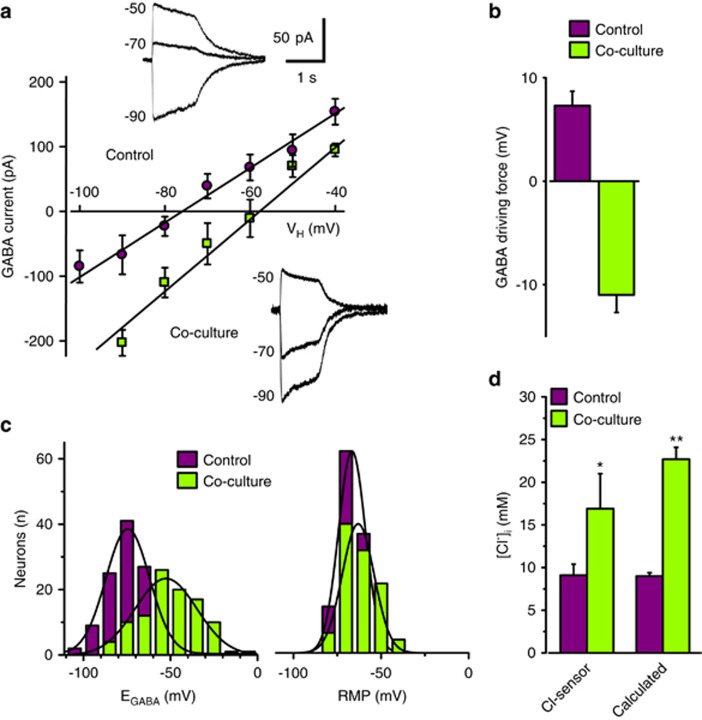
Glioma co-culture increases basal [Cl^−^]_i_ in mouse hippocampal neurons depolarizing E_GABA_. (**a**) Average I–V relation of GABA-activated currents recorded by perforated patch in control (*n*=24; purple circles) and glioma co-cultured (24 h,1 : 1 ratio; *n*=18; green circles) neurons (12 DIV); typical traces recorded in control and co-cultured neurons at indicated membrane potentials; (**b**) Left, distributions of E_GABA_ in control (purple columns) and co-cultured neurons (green columns; average E_GABA_, control −73.9±1.2 mV, co-culture −52.1±1.6 mV; *n*=124/101, *P*<0.001 ANOVA); Right, distributions of RMP in control (purple columns) and co-cultured neurons (green columns; average RMP, control −66.6±0.7 mV, co-culture −63.1±0.9 mV; *n*=124/101, *P*<0.05 ANOVA); (**c**) Driving force for GABA (RMP—E_GABA_) (control/co-culture as in (**b**); *n*=124/101; *P*<0.001 unpaired *t*-test); (**d**) basal neuronal [Cl^−^]_i_ measured in neurons transfected with Cl-Sensor (control, [Cl^−^]_i_=9.1±1.2 mM, purple column *n*=243; co-cultures, [Cl^−^]_i_ =16.9±4.1 mM; green column, *n*=43), and calculated using Nernst equation (control, *n*=124; co-culture, *n*=101; *P*<0.001 unpaired *t*-test)

To address the effect of glioma cells on neuronal Cl^−^ equilibrium, we determined the reversal potential of the currents evoked by GABA application in mouse hippocampal cultured neurons. As shown in Figures 1a and b, co-culturing neurons with patient-derived glioma cells (MZC)^14^ caused a rightward shift in the current–voltage relationship of GABA-mediated responses, giving a positive shift of E_GABA_ from −73.9±1.2 mV (control; *n*=124) to −52.1±1.6 mV (co-culture; *n*=101, *P*<0.001).

In control neurons, E_GABA_ was significantly below resting membrane potential (RMP); conversely, in co-cultured neurons, despite a small depolarization of resting potential (Figure 1b), E_GABA_ value was consistently more positive than RMP, reverting the driving force for GABA-mediated currents (Figure 1c). Similar results were observed also after a shorter (4h) co-culture duration (E_GABA_=−52.2±4.4 mV; *n*=18). E_GABA_ shift resulted from the increase in [Cl^−^]_i_, as directly demonstrated using a genetically encoded Cl-Sensor,^15, 16^ which gave values similar to those calculated by Nernst equation (Figure 1d).

**Figure 2:**
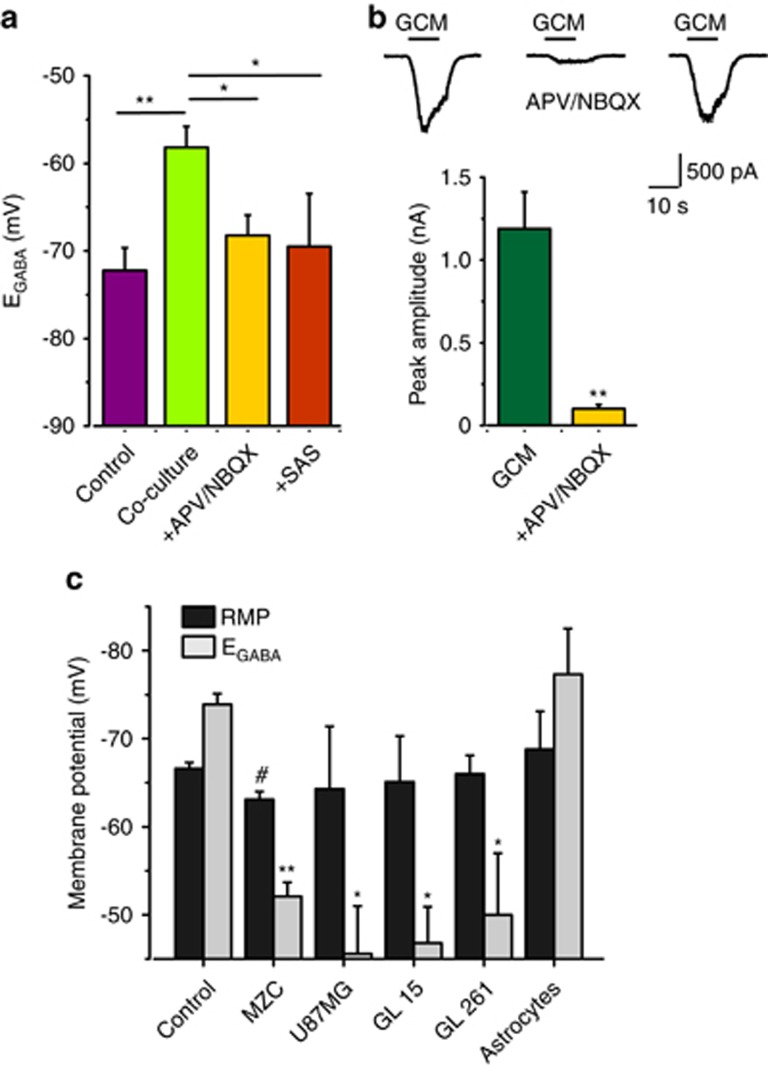
E_GABA_ depolarization is due to glutamate release by glioma cells. (**a**) Inhibition of GluRs or transport prevents E_GABA_ shift in co-cultured neurons; E_GABA_ in glioma co-culture either untreated (−58.2±2.4; *n*=35; green), in the presence of APV (20 *μ*M) and NBQX (10 *μ*M) (−68.2±2.3; *n*=27; *P*<0.05, ANOVA; yellow), or of Xc glioma glutamate transport system blocker, sulfasalazine (250 *μ*M; −69.5±6.0; *n*=10; *P*<0.05, ANOVA; orange) (control E_GABA_.−72.2±2.6; *n*=15; purple). (**b**) GCM activates glutamatergic currents; *Top*, currents elicited in hippocampal neurons by GCM are blocked by ionotropic glutamatergic antagonists (NBQX 10 *μ*M; APV 40 *μ*M) and recover upon drugs washout; *bottom*, average amplitude of GCM-induced current responses before and after treatment with NBQX/APV (*n*=14; yellow). (**c**) RMP (dark grey columns) and E_GABA_ (light gray columns) values in neurons co-cultured with different glioma cell lines; MZC (*n*=101), U87MG (*n*=7), GL15 (*n*=4), GL261(*n*=10) and astrocytes (*n*=7; *P<0.05, **P<0.01 *versus* control neurons, ANOVA). Notice that MZC co-culture caused slight RMP depolarization (# *P*<0.05). Data and the corresponding estimated [Cl^−^]_i_, calculated using Nernst equation, are reported in Supplementary Table S1

To investigate the possible role of glutamate released by glioma cells in E_GABA_ depolarization, glutamate receptors (GluRs) antagonists (D-(−)-2-Amino-5-phosphonopentanoic acid (APV), 20 *μ*M; 2,3-Dioxo-6-nitro-1,2,3,4-tetrahydrobenzo[f]quinoxaline-7-sulfonamide disodium salt (NBQX), 10 *μ*M) were added to the co-culture medium. In this condition, glioma-induced E_GABA_ depolarizing shift was prevented (Figure 2a). Similar effects were observed when co-culture experiments were performed in the sole presence of APV (E_GABA_=−70.6±1.96 mV; *n*=12, *P*<0.01) or NBQX (E_GABA_=−70.5±7.7; *n*=6, *P*<0.01), indicating that activation of both AMPA and NMDA receptors is necessary to cause E_GABA_ shift.

In addition, when the system Xc blocker sulfasalazine (250 *μ*M) was added to the co-culture medium, E_GABA_ depolarization was prevented (Figure 2a), showing that this effect requires Xc-mediated glutamate release.

Moreover, acutely applied glioma-conditioned medium (GCM) activated APV/NBQX-sensitive inward currents (Figure 2b) in hippocampal neurons, confirming the presence of GluRs agonists in GCM.^17^

Altogether, these data show that in the co-culture conditions, glioma cells cause the depolarizing shift of Cl^−^ equilibrium potential in neurons, through glutamate release and GluRs activation. Consistently, E_GABA_ depolarization was observed in neurons co-cultured with different human and murine glioma cells, but not with astrocytes (Figure 2c, and Supplementary Table S1), indicating a tumor-specific effect.

### Glioma-induced E_GABA_ depolarization is due to Cl^−^ transporters unbalance

**Figure 3:**
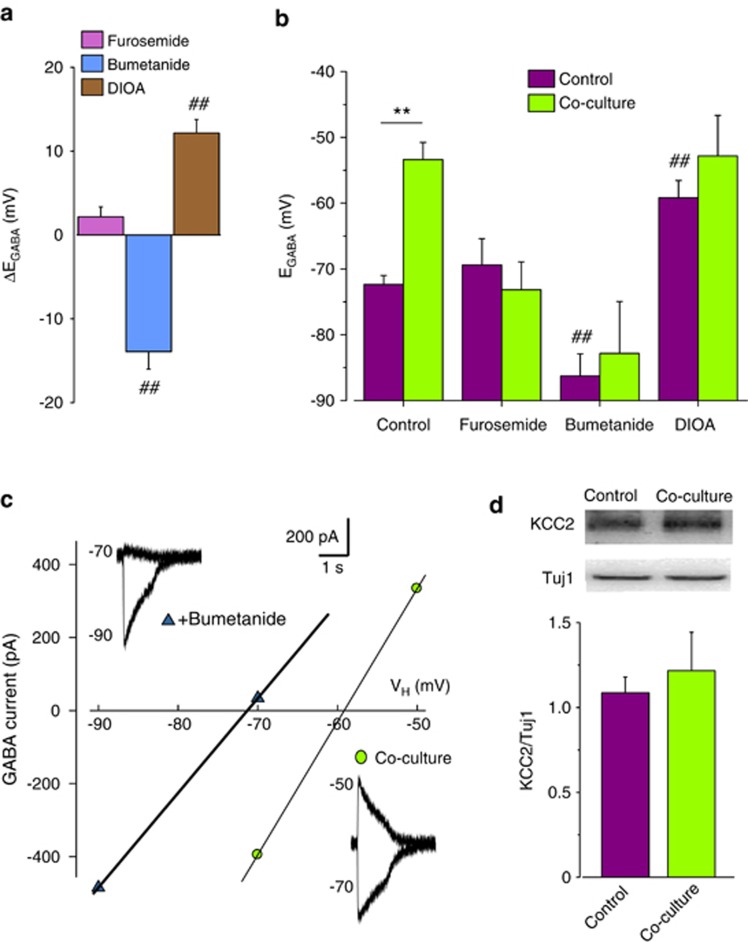
E_GABA_ depolarizing shift depends on Cl^−^ transporters unbalance. (**a**) E_GABA_ shift in 12 DIV cultured hippocampal neurons induced by acute application of KCC2 and NKCC1 blocker furosemide (100 *μ*M, 5–15 min; *n*=9; pink), NKCC1 blocker bumetanide (10 *μ*M, 5–15 min; *n*=12, ^##^*P*<0.01 respect to control; cyan) and KCC2 blocker DIOA (20 *μ*M, 1 h pre-application and during the experiment; *n*=13, ^##^*P*<0.01 respect to control; brown). Notice that furosemide, bumetanide or DIOA application was uneffective on neuronal RMP; (**b**) Cl^−^ transporters blockers abolish E_GABA_ shift induced by glioma co-culture in hippocampal neurons; effect of co-culture on E_GABA_ in neurons either untreated (control, −72.4±1.4; *n*=14; co-culture, −53.4±2.6; *n*=9; *P*<0.05) or treated with furosemide (control, −69.4±4; *n*=9; co-culture, −73.2±4.3; *n*=18; *P*=0.12), bumetanide (control, −86.3±3.4; *n*=12; co-culture, 82.8±7.9; *n*=9; ^##^*P*<0.01 respect to control) and DIOA (control, −59.2±2.6; *n*=13; co-culture, −52.8±6.1; *n*=11; ^##^*P*<0.01 respect to control) (unpaired *t*-test). (**c**) Acute bumetanide application reverts E_GABA_ shift in co-culture. Traces and I–V relation from a single co-cultured neuron before (purple circles) and following application of 10 *μ*M bumetanide (cyan triangles; 5–15 min, *n*=5). (**d**) Glioma co-culture does not alter neuronal KCC2 expression. *Bottom*, western blot analysis of KCC2 expression in hippocampal neurons in control and after glioma cell co-culture (n=6-9; ANOVA); top, representative immunoblots ofKCC2 (140  KDa) and *β*-tubulin III (Tuj1, 60 KDa) expression in control and co-cultured neurons as indicated

In cultured hippocampal neurons, KCC2 and NKCC1 transporters participate in the regulation of Cl^−^ homeostasis. Indeed, the acute application of furosemide (100 *μ*M; 5–15 min), the blocker of both transporters, caused a minor shift in neuronal E_GABA_. Conversely, the specific blockers of NKCC1 (bumetanide; 10 *μ*M; acutely applied 5–15 min) and KCC2 (R-(+)-butylindazone dihydroindenyl-oxy alkanoic acid, DIOA; 20 *μ*M; 1 h pre-treatment plus continuous superfusion) shifted E_GABA_ in opposite directions (Figure 3a), indicating that, in our culture conditions, both transporters actively regulate [Cl^−^]_i_.

The alteration of Cl^−^ transporter activity is involved in glioma-induced E_GABA_ shift in co-culture. In fact, in co-cultured neurons, furosemide, bumetanide or DIOA treatment abolished E_GABA_ depolarization (Figure 3b). However, the acute application of bumetanide reverted the glioma-induced effect (Figures 3b and c), hyperpolarizing E_GABA_. Conversely, DIOA treatment significantly depolarized E_GABA_ by itself, likely occluding co-culture depolarizing effect (Figure 3b). These data indicate that glioma-induced E_GABA_ depolarization is due to the unbalance of cation-chloride transporters activity, likely due to KCC2 reduced function.

To disclose the effects of glioma co-culture on neuronal KCC2 protein expression, we performed immunoblot experiments, revealing that the level of transporter expression was similar in control and co-cultured neurons (Figure 3d), thus indicating that glioma-induced alteration of neuronal Cl^−^ homeostasis relies on functional KCC2 modulation, rather than on changes in expression.

### Glioma co-culture causes KCC2 impairment by increasing intracellular Zn^2+^ in neurons

**Figure 4:**
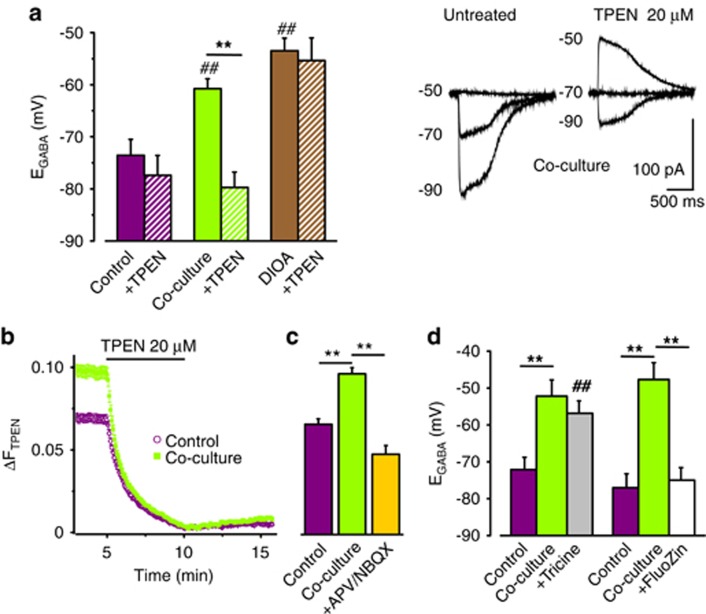
E_GABA_ shift is dependent on intracellular Zn^2+^ rise. (**a**) Co-culture-induced E_GABA_ depolarization depends on Zn^2+^-mediated KCC2 inhibition. *Left,* TPEN application reverts co-culture induced E_GABA_ shift (co-culture/TPEN, −60.8±1.9 mV; −79.7±3 mV; *n*=16, *P*<0.01; control/TPEN, −72.8±1.9 mV; −77.4±3.8 mV; *n*=14; *P*=0.76; paired *t*-test), but fails to rescue E_GABA_ depolarization when KCC2 is blocked by DIOA (20 *μ*M; DIOA −53.5±2.4 mV; plus TPEN −55.3±4.3 mV; *n*=6; *P*=0.78). *Right*, traces from a single co-cultured neuron before and following TPEN (20 *μ*M) treatment. (**b**) Co-cultured neurons have higher basal Zn^2+^. Fluorescence monitoring from FluoZin-3 loaded control (purple circles; *n*=125) and co-cultured (green squares; *n*=158) neurons exposed to TPEN. (**c**) Co-culture-induced intracellular Zn^2+^ rise (control, purple column, *n*=67; co-culture, green column, *n*=98) is prevented by GluRs blockers (APV 20 *μ*M; NBQX 10 *μ*M, *n*=24, yellow column ordinate as in **b**). (**d**) Effects of Zn^2+^ chelation on co-culture-induced E_GABA_ depolarization. E_GABA_ shift was absent when neurons were co-cultured in the presence of the Zn^2+^-sensitive dye FluoZin (5 *μ*M), chelating intracellular Zn^2+^ (control, −77±3.8 mV; *n*=8; 4 h co-culture −47.7±4.6 mV; *n*=13; 4h co-culture plus FluoZin −75±3.5 mV; *n*= 13; *P*<0.01), while tricine (1 mM, added to the co-culture medium, and washed during recordings, to avoid acute KCC2 inhibition mediated by extracellular Zn^2+^ removal^19^) was ineffective (control, −72.1±3.4 mV; *n*=13; 4h co-culture −52.2±4.4 mV; *n*=17; 4 h co-culture plus tricine −56.8±3.3 mV; *n*= 6; *P*=0.65; ^##^*P*<0.01 *versus* control cultures treated with tricine)

To investigate a possible role of intracellular Zn^2+^ on glioma-induced E_GABA_ shift,^18^ we used the membrane permeant Zn^2+^ chelator N,N,N′,N′-tetrakis(2-pyridylmethyl)ethylenediamine (TPEN). TPEN application (20 *μ*M; 5–20 min) caused the rapid recovery of neuronal E_GABA_ from glioma-induced depolarization (Figure 4a). This result suggests that glioma-induced E_GABA_ shift can be ascribed to KCC2 inhibition, caused by neuronal Zn^2+^ rise. Accordingly, when similar E_GABA_ depolarization was obtained blocking KCC2 with the selective antagonist DIOA (20 *μ*M; 1 h pre-treatment plus continuous superfusion), acute TPEN application had no effect (Figure 4a).

Using FluoZin-based fluorescence determinations, we also observed that basal intracellular Zn^2+^ was significantly higher in co-cultured neurons in respect to control (Figure 4b). Neuronal Zn^2+^ accumulation was prevented by APV/NBQX application in the co-culture medium (Figure 4c), indicating the requirement for GluR activation. Consistently, the application of GCM or glutamate (20 *μ*M) onto FluoZin-loaded neurons, elicited an APV/NBQX-sensitive fluorescence increase, rapidly reverting to basal level upon TPEN application (Supplementary Figure S1 and S2).

To identify the source of Zn^2+^, we performed experiments in the presence of tricine, a chelator of extracellular Zn^2+^. Tricine treatment (1 mM, 24 h) did not abolish the effect of co-culture on E_GABA_ (Supplementary Table S2), indicating that extracellular Zn^2+^ is not required for E_GABA_ depolarization. However, co-culture induced [Zn^2+^]_i_ accumulation was not observed (not shown). Noteworthy, tricine treatment caused a reduction in basal Zn^2+^ both in control and co-cultured neurons (*n*=41/59, control/co-culture; not shown). These results suggest that extracellular Zn^2+^ chelation modifies neuronal Zn^2+^ homeostasis, altering basal cytosolic Zn^2+^ level. To avoid possible effects on intracellular Zn^2+^ homeostasis, experiments with Zn^2+^ chelators were repeated reducing incubation time (4 h), following the observation that 4 h tricine treatment did not impair neuronal ability to release intracellular Zn^2+^ in response to a glutamatergic stimulus (Supplementary Figure S2). Consistently, tricine did not prevent the depolarizing effect of 4 h co-culture on E_GABA_ (Figure 4d). Conversely, when intracellular Zn^2+^ was chelated (with FluoZin 5 *μ*M, 4 h), co-culture-induced E_GABA_ shift was abolished (Figure 4d).

All together, these data indicate that Cl^−^ disequilibrium in co-cultured neurons is due to intracellular Zn^2+^-dependent KCC2 impairment.

### Zn^2+^-mediated E_GABA_ shift requires Src/TrkB activation

**Figure 5:**
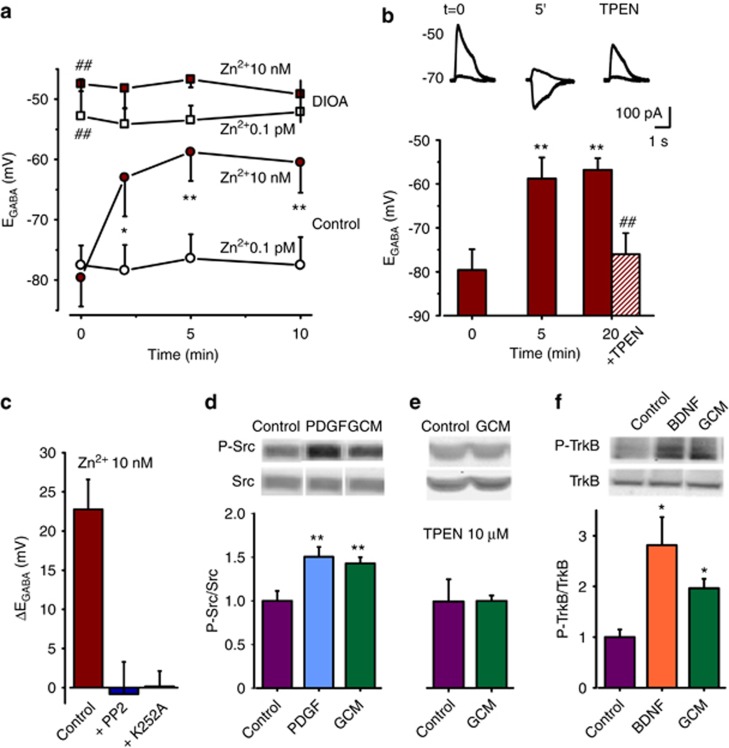
Intracellular Zn^2+^ modulates E_GABA_ through Src and TrkB activation. (**a**) *Bottom,* E_GABA_ is rapidly depolarized in cultured hippocampal neurons loaded with ~10 nM free Zn^2+^ through the patch pipette (dark red circles; *t*=0, −79.6±4.7 mV; t=5′, −58.8±4.8 mV; *n*= 17; *P<0.05; **P<0.01, paired *t*-test), but not with standard EGTA-containing intracellular solution (empty circles; free Zn^2+^=0.1 pM; *P*=0.54; *n*=8). *Top,* in cultures treated with the KCC2 blocker DIOA (20 *μ*M, 1 h pre-application and during the experiment), E_GABA_ was significantly depolarized at *t*=0 with both intracellular solutions (dark red squares, free Zn^2^ ~10 nM, −47.5±1.2 mV; *n*=5; empty squares, free^+^Zn^2+^=0.1 pM, −52.8±6.1 mV, *n*=6; ^##^*P*<0.01; respect to control, ANOVA ), and remained stable despite the intracellular Zn^2+^ loading. (**b**) TPEN application reverts Zn^2+^-induced E_GABA_ shift. *Top*, sample current traces from 10 nM free Zn^2+^-loaded neuron, at start of recording (*t*=0), 5′ after and following TPEN application (20 *μ*M; 15′). *Bottom*, Zn^2+^-induced E_GABA_ depolarization at *t*= 0; 5′ and 20′ (*n*=7, **P<0.01, ANOVA); dashed column represents E_GABA_ after 15′ TPEN application (following 5′ Zn^2+^ loading; *n*=8, ^##^*P*<0.01 *versus* 20′, ANOVA-Tukey test). (**c**) Impairment of Zn^2+^-induced E_GABA_ depolarization (ΔE_GABA_ at t=5′, 22.8±3.8 mV, *n*=9) in the presence of Src kinases inhibitor (PP2, 5 *μ*M, *n*=9, *P*<0.01) or TrkB inhibitor (K252A, 200 nM; *n*=7, *P*<0.01) (*versus* control, ANOVA). E_GABA_ and RMP were depolarized in control following, K252A or PP2 treatment as reported in Supplementary Table S4. (**d**) GCM increases Src phosphorylation. *Top*, typical immunoblot experiment; *bottom*, GCM treatment (15′) increases Src (*n*=10, *P*<0.01; ANOVA) phosphorylation (platelet-derived growth factor represent positive control). (**e**) TPEN application prevents GCM-induced Src phosphorylation; *Top*, typical immunoblot experiment; *bottom*, GCM treatment (15′) failed to increase Src (*n*=6, *P*=0.92; ANOVA) phosphorylation in cultures pre-treated with TPEN (20 *μ*M, 15′ *y-*axis as in (**d**). (**f**) GCM increases TrkB phosphorylation. *Top*, typical immunoblot experiment; *bottom*, GCM treatment (15′) increases TrkB (*n*=3, *P*<0.05; ANOVA) phosphorylation (BDNF represents positive control)

To disclose the mechanisms underlying Zn^2+^-mediated E_GABA_ shift, neuronal [Zn^2+^]_i_ was artificially increased through perforated (by gramicidin) patch pipette loading. We preliminarily verified the efficacy of intracellular Zn^2+^ loading through perforated patch by fluorescence recordings; the presence of ZnCl_2_ in the pipette solution caused a time- and concentration-dependent fluorescence increase in FluoZin-loaded neurons, indicating that, in our experimental conditions, Zn^2+^ permeates through gramicidin pores (Supplementary Table S3; Supplementary Figure S3). When 0.1 *μ*M ZnCl_2_ was added to a BAPTA (5 mM) containing intracellular solution, giving a controlled free [Zn^2+^]_i_ of ~10 nM (Supplementary Table S3) E_GABA_ promptly shifted to more depolarized values (Figures 5a and b). Conversely, E_GABA_ remained stable with control intracellular solution (calculated as~0.1 pM free [Zn^2+^]_i_, Figure 5a), or when free [Zn^2+^]_i_ was below 2 nM (*n*=7; Supplementary Table S3). This time-dependent E_GABA_ depolarization was not observed when cultures were treated with the KCC2 blocker DIOA (20 *μ*M; 1 h pre-treatment plus continuous superfusion; Figure 5a and Supplementary Table S4). In these conditions, neurons displayed a depolarized E_GABA,_ likely occluding the effects of intracellular Zn^2+^ loading. Consistently, acute TPEN application (15 min; Figure 5b) rapidly reverted Zn^2+^-induced E_GABA_ depolarization on control neurons. These data indicate that intracellular Zn^2+^ level rapidly and reversibly interferes with Cl^−^ equilibrium, through KCC2 activity modulation, highlighting intracellular Zn^2+^ rise as the key step in glioma-induced E_GABA_ depolarization.

Several mechanisms have been proposed to explain KCC2 downregulation in hyperexcitability models,^7, 20, 21^ including the phosphorylation of KCC2 residues by a number of different kinases. To explore the mechanism of Zn^2+^-mediated E_GABA_ shift, we investigated the involvement of Src/TrkB-dependent KCC2 tyrosine phosphorylation,^20, 21^ as intracellular Zn^2+^ has been reported to transactivate TrkB in a Src-dependent manner.^22^ When hippocampal cultures were treated with TrkB inhibitor K252A (200 nM; 1 h pre-application and perfused during the experiment), intracellular Zn^2+^ failed to depolarize neuronal E_GABA_ (Figure 5c and Supplementary Table S4). Similarly, in the presence of Src kinase inhibitor PP2 (5 *μ*M; 1 h pre-application and perfused during the experiment), E_GABA_ shift due to intracellular Zn^2+^ loading was absent (Figure 5c and Supplementary Table S4). Thus, Zn^2+^-induced E_GABA_ shift requires the integrity of Src/TrkB pathway.

Consistently, by western blots analysis, we demonstrated that GCM treatment (15 min) significantly increased neuronal Src phosphorylation (Figure 5d). This effect was Zn^2+^ dependent, as it was prevented by TPEN application (20 *μ*M, 15 min pre-treatment and during GCM application, *n*=6; *P*=0.92 with respect to TPEN, Figure 5e). Moreover, GCM treatment significantly increased neuronal TrkB phosphorylation (Figure 5f).

**Figure 6:**
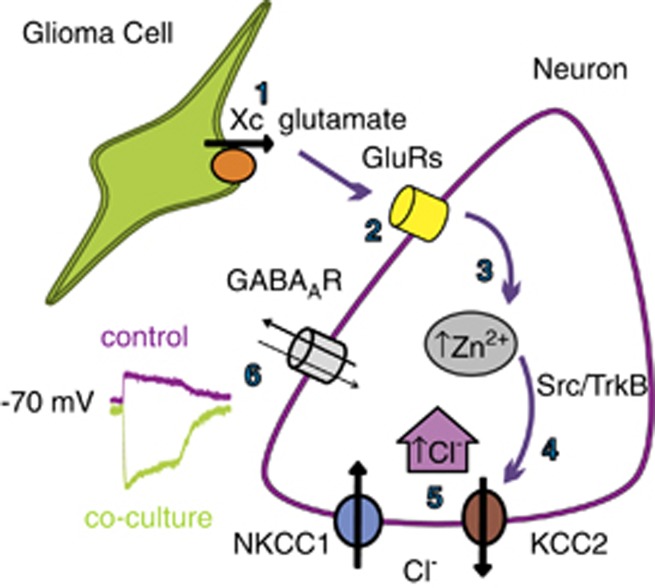
Model of glioma-induced E_GABA_ shift. Glutamate released from glioma cells (1) causes GluR-dependent (2) neuronal [Zn^2+^]_i_ increase (3), Src/TrkB-mediated KCC2 inactivation (4) leading to [Cl^−^]_i_ increase (5) and E_GABA_ shift (6)

Altogether, these data indicate that glioma-released factors might alter neuronal Cl^−^ homeostasis through Zn^2+^-induced Src/TrkB-mediated KCC2 modulation, as illustrated in Figure 6.

## Discussion

We used co-cultures of hippocampal neurons and glioma cells to unveil the mechanisms of glioma-induced hyperexcitability, reporting that glioma cells depolarize neuronal E_GABA_, increasing [Cl^−^]_i_ and reverting the driving force for GABA-mediated currents. Our results show that E_GABA_ depolarization relies on Zn^2+^-mediated KCC2 functional impairment, disclosing the underlying mechanism: glioma-released glutamate activates neuronal GluRs, causing neuronal intracellular Zn^2+^ rise which, through Src/TrkB activation, reduces KCC2 activity, leading to intracellular [Cl^−^] increase and E_GABA_ depolarization. We conclude that glioma might reduce neuronal inhibition through Zn^2+^-mediated downregulation of KCC2 activity, causing hyperexcitability.

In glioma-co-cultured hippocampal neurons, the current–voltage relationship of GABA-mediated responses is shifted to more depolarized potentials, compared with control, giving a more depolarized E_GABA_. This indicates a higher basal neuronal [Cl^−^]_i_ in co-cultures, confirmed by an independent estimation in neurons transfected with a YFP-based Cl^−^Sensor. Although glioma co-culture induces a small neuronal depolarization, the shift of E_GABA_ is more relevant, resulting in the inversion of the driving force for GABA-mediated currents.

According to previous studies, Cl^−^ homeostasis in cultured hippocampal neurons is determined by the activity of both NKCC1 and KCC2.^8^ Indeed, we show here that both transporters are expressed in control neurons and their activity is required to maintain Cl^−^ equilibrium as blocking either NKCC1 or KCC2 leads to a shift in basal E_GABA_. Conversely, in co-cultured neurons, NKCC1 activity is apparently not balanced by KCC2-mediated Cl^−^ extrusion and transporter disequilibrium leads to an increase in neuronal [Cl^−^]_i_. This picture is demonstrated pharmacologically by the use of selective antagonists of Cl^−^ co-transporters: (i) furosemide, the blocker of both transporters, abolishes E_GABA_ shift, demonstrating the involvement of Cl^−^ transport; (ii) the specific NKCC1 antagonist bumetanide reverts co-culture-induced E_GABA_ depolarization, demonstrating that Cl^−^ accumulation requires the activity of NKCC1; (iii) neurons treated with the specific KCC2 blocker DIOA display depolarized E_GABA_, likely occluding the co-culture effect. Altogether, these data support the proposal that in neurons, a new Cl^−^ equilibrium is established during glioma co-culture, characterized by higher [Cl^−^]_i_ and caused by reduced KCC2 activity.

We provide evidence that glioma-induced KCC2 impairment, observed in co-cultured neurons, is due to intracellular Zn^2+^ rise.^18^ Indeed, intracellular Zn^2+^ chelation by TPEN rapidly reverts co-culture-induced E_GABA_ shift and basal [Zn^2+^]_i_ is significantly higher in neurons after glioma co-culture.

The effect of neuronal Zn^2+^ rise on E_GABA_ was directly evaluated in experiments where intracellular [Zn^2+^] was artificially increased from pico to nanomolar, as in pathological conditions.^23, 24^ The resulting Zn^2+^-dependent E_GABA_ shift is ascribable to KCC2 impairment, because it was absent when KCC2 was pharmacologically blocked by DIOA. Indeed, the effect of Zn^2+^ on E_GABA_ is likely occluded by pharmacological KCC2 block, as DIOA treated neurons already show a depolarized E_GABA_, which is, consistently, not rescued by TPEN application.

Glioma-induced alteration of neuronal Cl^−^ homeostasis likely depends on functional KCC2 block, rather than on reduction of protein expression (see Lee *et al.*^7^). This view is based on the unaltered expression of neuronal KCC2 after glioma co-culture and on the rapid rescue exerted by Zn^2+^ chelation, demonstrating a dynamic modulation of Cl^−^ transport mechanisms. The functional modulation of KCC2 activity has been observed both in physiological and pathological models, such as prolonged post-synaptic spiking,^25^ brain-derived neurotrophic factor (BDNF) stimulation^26^ and oxygen glucose deprivation.^18^ Our data suggest that the mechanism responsible for glioma-induced KCC2 inhibition relies on Zn^2+^-mediated Src/TrkB activation. Indeed, Zn^2+^-induced E_GABA_ shift was prevented by the application of Src or TrkB kinase inhibitors (PP2 or K252A). Consistently, GCM increased the level of TrkB and Src phosphorylation in hippocampal cultures, the latter effect being prevented by the Zn^2+^ chelator TPEN. These results are in line with the notion that intracellular Zn^2+^ may transactivate TrkB by a neurotrophin-independent and Src-dependent mechanism, as reported in models of intense neuronal activity.^22^

In neurons, Zn^2+^-induced TrkB transactivation may mimic BDNF-TrkB signaling, leading to KCC2 phosphorylation on tyrosine residues^27, 28^ and driving neuronal disinhibition.^29^ We can speculate that co-culture induced E_GABA_ depolarization needs KCC2 phosphorylation on tyrosine residues because of Zn^2+^-induced TrkB transactivation.

We report that glioma-induced E_GABA_ shift requires the release of glutamate in the extracellular space by glioma cells and the consequent activation of neuronal ionotropic GluRs. Indeed, the application of APV and NBQX during co-culture prevents E_GABA_ depolarization.

It is known that glutamate can induce intracellular Zn^2+^ increase through different mechanisms, including AMPARs and Ca^2+^ channel-mediated influx or Ca^2+^-dependent intracellular release.^25, 30^ We observed that both APV and NBQX abolished co-culture-induced E_GABA_ depolarization highlighting the role for both ionotropic GluRs in this effect. The simplest explanation for this evidence is that AMPAR-mediated neuronal depolarization drives NMDAR activation thus allowing Ca^2+^-dependent intracellular Zn^2+^ release.^30^ Our data indicate that the source of Zn^2+^ is intracellular, as the extracellular Zn^2+^ chelator tricine was ineffective, whereas FluoZin prevented co-culture-induced E_GABA_ depolarization.

It has to be considered that tricine treatment, although did not prevent co-culture-induced E_GABA_ depolarization, inhibited co-culture-induced basal [Zn^2+^]_i_ accumulation. Thus, perturbing extracellular Zn^2+^ concentration may modify neuronal Zn^2+^ homeostasis, preventing cytosolic Zn^2+^ accumulation. However, tricine-treated neurons retained the ability to release intracellular Zn^2+^ in response to a glutamatergic stimulus, and this event is likely sufficient to trigger the intracellular signaling leading to KCC2 impairment.

It is well established that intracellular Zn^2+^ rise induces cell death, and Zn^2+^ exposure is toxic to neurons both *in vitro* and *in vivo*. It is now evident that increased cytosolic Zn^2+^ resulting from liberation from intracellular stores, rather than cytoplasmic influx of synaptically released Zn^2+^, can be highly toxic during oxidative and other types of neuronal injury,^31^ and Zn^2+^ dyshomeostasis appears to be a common feature of numerous neuropathological conditions.^23, 32^ We speculate that the reported mechanism, leading to reduced GABAergic transmission, could underlie the etiology of glioma-related epilepsy, pointing to Zn^2+^ accumulation as a possible therapeutic target to restore KCC2 function and the excitatory/inhibitory balance. In this view, it is possible to speculate that Zn^2+^ homeostatic drugs may be helpful in the treatment of Zn^2+^-related neurological disorders such as neuronal hyperexcitability or Alzheimer's disease.^33^

The use of co-cultures^34^ allowed to disclose the molecular mechanisms involved in glutamate-mediated overexcitation induced by glioma. Our results are in line with recent works showing that Xc-mediated glutamate release is responsible for the generation of tumor-associated epileptic events in glioma-bearing mice.^6, 35^ Indeed, increased concentration of extracellular glutamate has been found in peritumoral tissue in both humans and mice,^6, 36^ supporting its role in tumor growth, survival and peritumoral seizure activity.^6^ Additional sources of glutamate in peritumoral tissue may be microglial Xc system or reverse activity of Na^+^-dependent glutamate transporters in neurons or astrocytes.^37, 38^ Thus, the co-culture system likely retains the feature of excessive glutamate release typical of glioma. Consistently, neuronal E_GABA_ shift was observed in co-culturing neurons with different glioblastoma cell lines, but not with astrocytes, supporting the view of a tumor-specific effect.^6^

Glutamate-induced alteration of neuronal Cl^−^ homeostasis may act concomitantly with other mechanisms, including the direct depolarizing effect of glutamate on neurons, displacing the excitation/inhibition balance toward an increased network excitability, thus promoting seizure onset.^35^

In conclusion, our study provides a possible explanation of the mechanisms by which glioma cells affect neuronal Cl^−^ equilibrium, highlighting the role of Zn^2+^, recently emerged in a variety of excitotoxic conditions, such as epilepsy, ischemia and brain trauma.^32^

## Materials and Methods

### Animals

Procedures using laboratory animals were in accordance with the international guidelines on the ethical use of animals from the European Communities Council Directive of November 24, 1986 (86/609/EEC). All efforts were made to minimize animal suffering and to reduce the number of animals used in accordance with the European Communities Council Directive of September 20, 2010 (2010/63/UE).

### Primary hippocampal neuronal cultures

Hippocampal neuronal cultures were prepared from newborn (P0-P1) C57BL/6 mice of either sex (Charles River - Research Models and Services, Lecco, Italy). In brief, after careful dissection from diencephalic structures, the meninges were removed and the hippocampi were chopped and digested in 1.25 mg/ml trypsin for 20 min at 37 °C. Cells were mechanically dissociated and plated at a density of 10^5^ in poly-L-lysine-coated glass coverslip (12 mm diameter) in serum-free Neurobasal Medium (Gibco Life Science, Life Technologies Italia, Monza, Italy), supplemented with B27 2 mM L-glutamine and 100 *μ*g/ml gentamicin (neuronal culture medium). Then, cells were kept at 37 °C in 5% CO_2_ for 10–13 days with medium replacement (1 : 1 ratio) three times per week. With this method, we obtained cultures composed by 60–70% neurons, 30–35% astrocytes and 4–5% microglia, as determined with *β*-tubulin III, glial fibrillary acidic protein and isolectin IB4 staining.^38^ The same procedure was followed to prepare rat hippocampal culture used for some immunoblot experiments.

### Glial primary cultures

Primary cortical glial cells were prepared from P0-P2 mice. Cerebral cortices were chopped and digested in 30 U/ml papain for 40 min at 37 °C and gently triturated. The dissociated cells were washed, suspended in Dulbecco's Modified Eagle Medium (Gibco, Life Technologies Italia) with Glutamax with 10% fetal bovine serum (Invitrogen, Life Technologies Italia) and plated at a density of 9–10 × 10^5^ in 175 cm^2^ cell culture flasks. At confluence (10–14 days *in vitro*, DIV), glial cells were shaken for 2 h at 37 °C to detach and remove microglial cells. These procedures gave almost pure astrocytes cell population (4–6% of microglia contamination), as verified by staining with glial fibrillary acidic protein and isolectin IB4.^34^

### Neuronal culture transfection

Hippocampal neuronal cultures were transfected at 9–10 DIV. One day before transfection, 50% of the culture medium was replaced with fresh medium. For transfection, 100 *μ*l of Neurobasal media were mixed with 2 *μ*l of NeuroMag (OZ Bioscience, Marseille, France) and 1 *μ*g of Cl-Sensor cDNA.^15, 16^ The mixture was incubated for 15–20 min at room temperature and thereafter distributed dropwise over the neuronal culture. Neuronal cultures were placed on a magnetic board (OZ Bioscience) and incubated for 15 min (37 °C, 5% CO_2_). One hour later, 50% of neuronal culture medium was substituted with fresh neuronal culture medium. Cells were used for experiments 24–76 h after transfection.

### Glioma cell culture

Patient-derived glioma cells (MZC; kindly provided by Dr. Antonietta Arcella, Neuromed, Italy) within passage 40,^14, 39^ glioblastoma cell lines: GL15,^14, 40^ U87MG (American Type Culture Collection, Rochville, MD, USA) and GL261^41^ were grown in Dulbecco's Modified Eagle Medium with Glutamax supplemented with 10% heat-inactivated fetal bovine serum, 100 U/ml penicillin G, 2.5 *μ*g/ml amphotericin B and 100 *μ*g/ml streptomycin at 37 °C in a 5% CO_2_ humidified atmosphere. Medium was changed three times per week; cells were used between 20th and 40th passage.

### Co-cultures

Glioma cells or astrocytes, prepared as above, were re-suspended in neuronal culture medium and replated by seeding 10^5^ cells onto 0.33 cm^2^ transwell cell-culture inserts (pore size 0.4 *μ*M, Corning B.V. Life Sciences, Amsterdam, The Netherlands), allowing traffic of small diffusible substances, but preventing cell-to-cell contact. Transwell inserts were transferred into 24-well cultures plates, containing 10–12 DIV primary hippocampal cultures in neuronal culture medium.

Neuronal viability after 4 and 24 h of co-culture was evaluated as reported in Supplementary Figure S4.

### Patch-clamp recordings

Patch-clamp recordings were obtained using glass electrodes (3–5 MΩ) filled with the following intracellular solution (in mM): 140 KCl, 2 MgCl_2_, 10 HEPES, 2 MgATP, 0.5 EGTA; pH 7.3, with KOH. Perforated patch-clamp recordings with access resistances between 30 and 40 MΩ were obtained using gramicidin D.^42^ Gramicidin, prepared every 2 h, was added to the pipette solution to a final concentration of 50 *μ*g/ml. During experiments, neurons were continuously superfused with normal extracellular solution containing (in mM): 140 NaCl, 2.5 KCl, 2 CaCl_2_, 2 MgCl_2_, 10 HEPES-NaOH and 10 glucose (pH 7.3), added with tetrodotoxin (0.2 *μ*M), using a gravity-driven perfusion system, consisting of independent tubes for standard and agonist-containing solutions, connected to a fast exchanger system (RSC-100; Bio-Logic, Claix, France). All recordings were performed at 24–25 °C. For I–V experiments, neurons were voltage-clamped at −70 mV with 6-s steps to each test potential; GABA (100 *μ*M; 500 ms; every 30 s) was applied 1 s after the voltage step onset. A linear regression was used to calculate the voltage dependence of GABA-evoked current. E_GABA_ was taken as the intercept of this best-fit line for each cell. We minimized bicarbonate flux through the GABA_A_ channels, using an HEPES-buffered extracellular solution (nominally CO_2_- and bicarbonate-free^43, 44^), so that E_GABA_ was an estimation of E_Cl_. Membrane currents, recorded with a patch-clamp amplifier (Axopatch 200B; Molecular Devices, Foster city, CA, USA), were filtered at 2 kHz, digitized (10 kHz) and acquired with Clampex 10 software (Molecular Devices). The stability of the patch was checked by repetitively monitoring the input and series resistance during the experiment, and recordings were discarded when any of these parameters changed by >10%.

### Zn2+ loading through gramicidin pores

When indicated (Figure 5 and Supplementary Figure S3), intracellular [Zn^2+^] was artificially increased in neurons through the perforated patch, using a BAPTA-based intracellular solution (in mM: 140 KCl, 2 MgCl_2_, 10 HEPES, 2 MgATP, 5 BAPTA) supplemented with ZnCl_2_ (0.01–100 *μ*M). The corresponding free Zn^2+^ concentrations were calculated using the MaxChelator software: http://www.stanford.edu/~cpatton/maxc.html, and are reported in Supplementary Table S3.

### Zn^+2^ imaging

Fluorescence images were acquired at room temperature (24–25 °C) using a customized digital imaging microscope. Excitation of fluorophores at various wavelengths was achieved using a 1-nm-bandwidth polychromatic light selector (Till Polychrome V), equipped with a 150 W xenon lamp (Till Photonics, Hillsboro, OR, USA). Fluorescence was visualized using an upright microscope (Axioskope) equipped with a 40x water-immersion objective (Achroplan CarlZeiss, Oberkochen, Germany) and a digital 12 bit CCD camera system (SensiCam, PCO AG, Kelheim, Germany). All the peripheral hardware control, image acquisition and image processing were achieved using customized software Till Vision v. 4.0 (Till Photonics). Changes in the intracellular Zn^2+^ level were monitored using the high-affinity Zn^2+^-sensitive indicator FluoZin-3AM (Invitrogen). Neurons were loaded by incubating coverslips for 45 min at 37 °C in 1 ml of HEPES-buffered salt solution containing: 120 mM NaCl, 5.4 mM KCl, 1.8 mM CaCl_2_, 0.8 mM MgCl_2_, 20 mM HEPES-NaOH and 15 mM glucose (pH 7.3), plus 5 mg/ml of bovine serum albumin and 5 *μ*M of FluoZin-3AM. For time-lapse recordings, FluoZin-3AM was excited at 480 nm (emission filter D535/40 nm, dichroic mirror 505DCLP). Zn^2+^ fluorescence changes are presented as ΔF/F_0_=(F−F_0_)/F_0_, or ΔF_TPEN_=(F_0_−F_TPEN_)/F_TPEN_, where F is the current fluorescence intensity, F_0_ is the fluorescence intensity before agonist application and F_TPEN_ represents fluorescence intensity after 5 min of TPEN application.

### Cl^−^ imaging

For non-invasive monitoring of [Cl^−^]_i_, cells were transfected with CFP/YFP-based Cl-Sensor^15^ and the optic system equipped with an emission filter D535/40 nm and a dichroic mirror 505DCLP (505 nm) was used. Cells expressing Cl-Sensor were excited at 445 and 485 nm wavelengths alternatively (50 ms, 0.1 Hz). [Cl^−^]_i_ changes are expressed as a ratio (R) of background-subtracted F445 and F485 (R=F445/F485). (Supplementary Figure S5) Calibration of Cl-Sensor (Supplementary Figure S6) was performed as in Markova *et al.*^15^ and Wassem *et al.*^16^ External solutions with different Cl^−^ concentrations (in mM: 1, 3, 7, 12, 20, 35, 50, 70, 82.4, 130, 164.8) were obtained by mixing the following solutions (in mM): (i) 164.8 KCl, 10 D-glucose, 20 HEPES, pH 7.32 and (ii) 164.8 K-gluconate, 10 D-glucose, 20 HEPES, pH 7.32. To increase the permeability of the cell membrane to Cl^−^ ions, *β*-escin (80 *μ*M, Sigma, St Louis, MO, USA) was added to the extracellular solution and applied during 2–5 min. Fluorescence signals corresponding to rising Cl^−^ concentrations were monitored from single Cl-Sensor expressing neurons, after *β*-escin removal.

### Glioma-conditioned medium (GCM)

GCM was prepared as follows: when cultures of glioma cells became confluent (~3 × 10^6^ in 10 cm culture dishes), the culture medium was substituted with 10 ml of filtered normal extracellular solution. The conditioned medium (GCM) was collected after incubating for 4 h, centrifuged, pH adjusted to 7.35 and then used for electrophysiological and imaging experiments.^17^ For immunoblot analysis of TrkB and Src activation, GCM was obtained incubating (4 h) glioma cells in Locke's buffer (in mM: 154 NaCl, 5.6 KCl, 3.6 NaHCO_3_, 2.3 CaCl_2_, 1 MgCl_2_, 5.6 glucose, 5 HEPES; pH 7.4).

### Cell stimulation and western blot analysis

For western blot experiments, 4 × 10^5^ hippocampal cells were plated on poly-L-lysine coated 12-well cultures plates.^45^

For the analysis of TrkB and Src activation, cultured hippocampal neurons were incubated for 2 h in Locke's buffer and stimulated for 15 min with either GCM or drugs: BDNF (100 ng/ml), platelet-derived growth factor (100 ng/ml). To assess the role of Zn^2+^ rise in Src phosphorylation, hippocampal cultures were treated with the Zn^2+^ chelator TPEN (20 *μ*M, 15 min pre-application, and during GCM stimulation). Corresponding cell lysates were run on SDS-polyacrylamide gel and analyzed for Src or TrkB phosphorylation. Densitometric analysis was performed with QuantityOne software (Bio-Rad Laboratories S.r.l., Segrate (MI), Italy) and phosphoprotein levels were normalized for TrkB and Src expression. For KCC2 expression determination, western blot analysis was performed on rat hippocampal cell lysates in control conditions and after 24 h co-culture with equal number of glioma cells. For each condition, equal amounts of proteins were loaded on SDS-PAGE gel for immunoblot analysis with rabbit anti-KCC2. Protein levels were normalized for TuJ1 expression.

### Drugs

GABA, Bicuculline methochloride, Tetrodotoxin citrate, NBQX and D-APV were purchased from Abcam Biochemicals (Cambridge, UK); Furosemide (100 mM in dimethyl sulfoxide (DMSO)), Bumetanide (50 mM in EtOH) and PP2 (10 mM in DMSO) were purchased from Tocris Biosciences (Bristol, UK); FluoZin-3AM (Invitrogen, 5 mM DMSO); K252a (Calbiochem, Merck S.p.a., Milano, Italy); DIOA (Santa Cruz Biotechnology, Inc., Heidelberg, Germany; 200 mM in EtOH); BDNF (PeproTech EC Ltd., London, UK); TPEN (10 mM in EtOH), Gramicidin (50 mg/ml in DMSO), Sulfasalazine (500 mM in DMSO), platelet-derived growth factor and all chemicals used were purchased from Sigma-Aldrich, Milan, Italy. Where not indicated, all drugs were dissolved in water. Antibodies were purchased and used as follows: rabbit anti-Phospho TrkB (phospho Y515, 1 : 500, Abcam, Cambridge, UK), rabbit anti-Phospho Src (phospho Y416, 1 : 2000, Abcam), mouse anti- Neuronal Class III *β*-tubulin (TuJ1, 1 : 2000, n.cat MMS-435P, Covance, San Diego, CA, USA), rabbit anti-actin (1 : 5000, Sigma-Aldrich); rabbit anti-KCC2 (1 : 1000; Millipore, Temecula, CA, USA).

### Drug Application

During electrophysiological and fluorescence measurements, neurons were continuously superfused with normal extracellular solution, using a gravity-driven perfusion system, consisting of independent tubes for standard and agonist-containing (GABA, GCM) solutions, connected to a fast exchanger system (RSC-100; Bio-Logic) positioned 50–100 *μ*m from the cell. Antagonists were usually acutely applied through a parallel tubes of the same perfusion system. DIOA, K252A and PP2 were pre-incubated for 1 h (37 °C) and then continuously applied during the experiments. Sulfasalazine, APV/NBQX, BAPTA-AM and tetrodotoxin citrate were applied during co-culture in the co-culture medium and not superfused during recordings (24 h). The values of EGABA and RMP of control neurons, following different drug treatments are reported in Supplementary Table S2 and Supplementary Table S4.

### Data analysis

Data, analyzed offline, are presented as mean±S.E.M.; we used the QuantityOne (Bio-Rad Laboratories S.r.l.) program for the densitometric analysis of all immunoblots. Origin 7 (Origin software; Microcal Software, Northampton, MA, USA) and Sigmaplot 11 (Systat Software Inc, London, UK) software were used for statistical analysis. Paired and unpaired *t*-test and one-way ANOVA were used for parametrical data, as indicated; Tukey test was used as *post hoc* test; Mann and Withney test for non parametrical data. We constructed I–V plots, cumulative distribution plots and fitted data points by linear or non-linear regression analysis using Origin software. Statistical significance for cumulative distributions was assessed with Kolmogorov-Smirnov test.

## Abbreviations

E_GABA_: Reversal potential of GABA-evoked currents
system Xc: glutamate-cystine transporter
APV: D-2-amino-5-phosphonopentanoic
[Cl-]I: chloride concentration
DMSO: dimethyl sulfoxide
GABA: *γ*-aminobutyric acid
GCM: glioma-conditioned medium
Glu: glutamate
NBQX: 2,3-dihydroxy-6-nitro-7-sulfamoyl-benzo[f]quinoxaline-2,3-dione
RMP: resting membrane potential
DIOA: R-(+)-butylindazone dihydroindenyl-oxy alkanoic acid
TPEN: N,N,N′,N′-tetrakis(2-pyridylmethyl)ethylenediamine
