# Supplementary material for: A role for intracellular zinc in glioma alteration of neuronal chloride equilibrium
Source: Cell Death Dis. 2014 Oct 30;5(10):e1501–. doi: 10.1038/cddis.2014.437 (PMC4237258; doi:10.1038/cddis.2014.437)
Supplement: Supplementary Figures [file cddis2014437x1.doc]

**Supplementary MATERIAL**

**Di Angelantonio et al.**

**Effect of co-colture on neuronal RPM, E_GABA_ and [Cl^-^]_i_.**

The effect of neuronal co-culture with different glioma cell lines and astrocytes, on neuronal E_GABA_ and resting membrane potential (RMP), and the corresponding estimated [Cl^-^]_i_, calculated using Nerst equation, are reported in **Supplementary Table S1**.

**Supplementary Table S1**.

|  | **RMP** | **E_GABA_ (mV) (n)** | **[Cl^-^]_i_ (mM)** |
| --- | --- | --- | --- |
| Control neurons | -66.6 ± 0.7 | -73.9 ± 1.2 (124) | 9.0 ± 0.4 |
| MZC co-cultured neurons | -63.1 ± 0.9* | -52.1 ± 1.6 (101) ** | 22.7 ± 1.4 ** |
| U87MG | -64.3 ± 7.1* | -46.0 ± 14 (7) * | 25 ± 7 * |
| GL15 | -65.1 ± 5.2 | -46.8 ± 4.1 (4)* | 24.3 ± 0.8 * |
| GL261 | -66.0 ± 2.1 | -50.0 ± 7 (10) | 23.4 ± 3.2* |
| Astrocytes | -68.8 ± 4.3 | -77.3 ± 5.2 (7) | 7.4 ± 0.9 |

*p<0.05; **p<0.01 respect to control neurons (E_GABA_, [Cl^-^]_i_)

**Supplementary Figure S1.**

**Supplementary Figure S1. GCM elicits a glutamatergic increase in intracellular zinc**

Application of GCM increases intracellular Zn^2+^ in neurons by glutamatergic mechanisms. *Left*, fluorescence trace from a sample neuron, showing the effect of GCM application, alone and in the presence of APV/NBQX. *Right***,** average fluorescence response elicited by GCM in the presence of GluR antagonists (% of control; n=13 neurons).

**Effect of drug treatments on RPM and E_GABA_, of control neurons**

**Supplementary Table S2.**

| **Treatment** | **E_GABA_ (mV)** | **RMP (mV)** |
| --- | --- | --- |
| APV+NBQX | -71.0 ± 2.2 (10) | -69.5 ± 6.1 |
| SAS | -73.5 ± 5.2 (5) | -67.3 ± 5.0 |
| Furosemide | -69.4 ± 4.1 (9) | -70.2 ± 4.2 |
| Bumetanide | -86.2 ± 3.4 (12)** | -68.9 ± 5.3 |
| DIOA | -55.4 ± 3.1 (19)** | -70.8 ± 5.8 |
| TTX | -74.0 ± 6.0 (8) | -66.8 ± 4.9 |
| TPEN | -77.4 ± 3.8 (14) | -71.4 ± 3.3 |
| TPEN + DIOA | -55.3 ± 4.3 (6)** | -67.6 ± 4.8 |
| Tricine | -70.1 ± 5.7 (7) | -58.2 ± 3.2 |

** p<0.01. Statistical significance was determined for each experimental condition in a parallel group of control neurons.

**Supplementary Figure S2**

**Supplementary Figure S2. Glutamate induced intracellular Zn^2+^ rise is not prevented by tricine treatment.** Average fluorescence response elicited by the application of glutamate (20 µM) in the presence of tricine (1 mM) in control (black, n=9) and tricine-treated neurons (grey, n=13).

**Zn^2+^ permeability through gramicidin pores**

In order to assess Zn^2+^ permeability through gramicidin pores, we loaded hippocampal cultures with FluoZin3-AM and monitored fluorescence during gramicidin perforated patch-clamp recordings, using intracellular solution containing different free Zn^2+^ concentrations. (Supplementary Table S3)

**Table S3. Free Zn^2+^** concentrations in intracellular solution

| **[ZnCl_2_], µM** | **Chelator, mM** | **Free Zn^2+^, nM** | **E_GABA_ (mV) (n)** |
| --- | --- | --- | --- |
| 0 # | EGTA 0.5 | 0.0001 * | -77.5 ± 4.6 (8) |
| 0.01 | BAPTA 5 | 1.7 * | -75.8 ± 4.2 (7) |
| 0.1 | BAPTA 5 | 9.4 * | -60.5 ± 5.2 (9) |
| 1 | BAPTA 5 | 86 |  |
| 100 | BAPTA 5 | 9200 |  |

# control intracellular solution

* Theoretical, we took into account that contaminating [Zn^2+^] in physiological solutions is typically in the order of 10 nM^44^.

**Supplementary Figure S3.**

**Supplementary Figure S3. Zn^2+^ permeates through gramicidin pores.** *Left*, in neurons recorded with intracellular solution containing 100 µM ZnCl_2_ , 5 mM BAPTA, and 50 µg/ml gramicidin, (free Zn^2+^ ~ 10 µM), FluoZin fluorescence rapidly increased indicating intracellular Zn^2+^ accumulation (n= 6, filled circles). Conversely, when neurons were recorded in cell-attached configuration (no gramicidin), using a pipette containing the same concentration of ZnCl_2_ (100 µM) and BAPTA (5 mM), FluoZin fluorescence remained stable (n=5, empty circles). *Right*, bar chart representing mean FluoZin fluorescence increase in neurons recorded with pipette solution containing gramicidin and rising concentration of ZnCl_2_  and different chelators (ZnCl_2_ = 0, n=6; ZnCl_2_ = 0.1 µM, n= 7; ZnCl_2_ = 1 µM, n= 4; ZnCl_2_ = 100 µM, n= 6) after 5 min of recording.

**Effect of drug treatments on RMP and E_GABA_ of neurons loaded with different ZnCl_2_ concentration.**

The effect of TrkB and Src kinase inhibitors on RMP and E_GABA_ of neurons loaded with different ZnCl_2_ concentration is reported in Supplementary Table S3. E_GABA_ Values are considered after 10 minutes of perforated patch recording.

Supplementary Table S4.

|  | **0.1 pM free Zn^2+^** | | **10 nM free Zn^2+^** | |
| --- | --- | --- | --- | --- |
|  | **E_GABA_ (mV)** | **RMP (mV)** | **E_GABA_ (mV)** | **RMP (mV)** |
| NES | -79.6 ± 4.7 (7) | -70.3 ± 4.5 | -58.8 ± 4.8 (9) | -67.7 ± 5.2 |
| DIOA | -55.4 ± 3.1 (19)** | -70.8 ± 5.8 | -46.8 ± 1.3 (4) | -61.8 ± 2.5 |
| K252A | -66.2 ± 3.1 (11) | -62.5 ± 7.1 | -63.2 ± 1.3 (7) | -63.2 ± 5.5 |
| PP2 | -63.7 ± 3.4 (8) | -60.3 ± 4.2 | -64.5 ± 3.1 (9) | -61.1 ± 4.7 |

** p<0.01 respect to control neurons.

**Supplementary Figure S4**

**Supplementary Figure S4. Neuron viability after glioma cell co-culture**

Excitotoxicity experiments were performed co-culturing hippocampal neurons with MZC in a transwell system for 4 or 24 hours. To evaluate neuron viability cells were treated with detergent-containing buffer (0.05% ethyl hexadecyl dimethylammonium bromide, 0.028% acetic acid, 0.05% Triton X-100, 0.3 mM NaCl, 0.2 mM MgCl_2_, in PBS pH 7.4) and counted in a hemacytometer as already described ^45^. Results are expressed as % of cell survival, taking as 100% untreated cells in control conditions (n=4).

**Supplementary Figure S5**


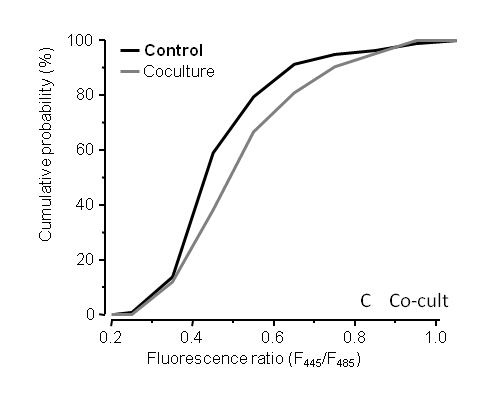


**Supplementary Figure S5.** **Ratio distributions in Cl-sensor-transfected neurons.**

Cumulative distributions of fluorescence ratios (445/485 nm) in co-cultured neurons (0.566 ± 0.025, n=43; thin line) vs control (0.515 ± 0.008, n=243; thick line), (p<0.05, Kolmogorov-Smirnov test).

**Supplementary Figure S6**

**Supplementary Figure S6. Calibration of Cl-Sensor.**

Each data point represents the mean ± SEM of R (6-25 neurons). Data were best fitted using the following logistic equation:


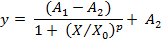


Where:

A_1_=R_min_, A_2_= R_max_, x_0_=[Cl^-^] at y=R_max_/2, p= n_H_. Notice that R_max_ was estimated at maximal [Cl^-^], 164.8 mM.

The calibration curve function was used to determine basal [Cl^-^]_i_ in neurons, fitting the mean ratio values of control and co-cultured neurons into the curve; SEM was calculated using error propagation function.
